# Ectopic Liver Tissue Mistakenly Diagnosed as a Right Atrial Myxoma

**DOI:** 10.7759/cureus.3862

**Published:** 2019-01-10

**Authors:** Mohanad Soliman, Olalekan Akanbi, Amr Salem, Mahmoud Khreis, Ahmed Abdel-Latif

**Affiliations:** 1 Internal Medicine, University of Kentucky School of Medicine, Lexington, USA; 2 Internal Medicine, Baystate Medical Center, Springfield, USA; 3 Cardiology, University of Kentucky School of Medicine, Lexington, USA

**Keywords:** atrial mass, abberant, malignant transformation, cardiac mri, spect, herniation

## Abstract

Myxomas, metastatic tumors, thrombi, and vegetations top the differential diagnosis list of cardiac masses. We present a case of ectopic liver tissue, a far less common etiology of a right atrial mass, discovered incidentally on transthoracic echocardiography (TTE) of a 37-year-old female with multiple comorbidities who was referred to our facility for further management of left popliteal artery occlusion and right lower extremity cellulitis. We discuss further the categories, proposed pathogenesis, diagnostic approach, and potential complications of ectopic liver tissue.

## Introduction

Aberrant liver tissue is an exceedingly rare condition that may have clinical implications ranging from mechanical compression to malignant transformation [1]. Typically, the aberrant hepatic tissue is found in the abdominal organs; however, it has also been described in the thoracic cavity [2]. In extremely rare occasions, ectopic hepatic tissue can present as a right atrial mass. Fujimoto and colleagues [3] described the first case of intracardiac aberrant liver tissue mistakenly diagnosed as a right atrial tumor in 1998.

## Case presentation

A 37-year-old Caucasian female with a past medical history significant for spina bifida complicated by paraplegia and neurogenic bladder, kyphoscoliosis with multiple spine surgeries, hypertension, sinus tachycardia, depression, and multiple episodes of right lower leg cellulitis presented as a transfer to our tertiary medical center for further management of a left popliteal artery occlusion and right lower extremity cellulitis. Her daily medications were trazodone, Celexa, tolterodine, propranolol, Wellbutrin, and hydrochlorothiazide.

On physical examination, she was calm, cooperative, and in no acute distress. She weighed 65 kg and was 134 cm tall. Her blood pressure was 119/85 mm Hg, heart rate 101 per minute, temperature 98.8°F, respiratory rate 18 per minute, and arterial oxygen saturation of 96% on 2 liters nasal cannula. Head and neck examination was normal, and she had moist oral mucosa with no oral thrush or ulcers noted. There were no abnormal lung or heart sounds. The abdomen was soft, non-distended, non-tender with no rebound or guarding, and normal bowel sounds. Physical examination revealed kyphoscoliosis of the back, underdeveloped paraplegic lower extremities with bilateral sensory loss, and intact distal pulses in the left foot with warm pink toes. The right lower leg and foot were swollen, erythematous, and non-tender with bullae of serosanguinous fluid. Her laboratory tests were normal, except for hypokalemia, with a potassium level of 2.4 mmol/L and lactic acid of 1.6 mmol/L.

The vascular surgery team was consulted. She was started on a heparin drip for left popliteal artery thromboembolism and the recommendation was made for transthoracic echocardiography (TTE) and antiphospholipid syndrome screening.

**Figure 1:**
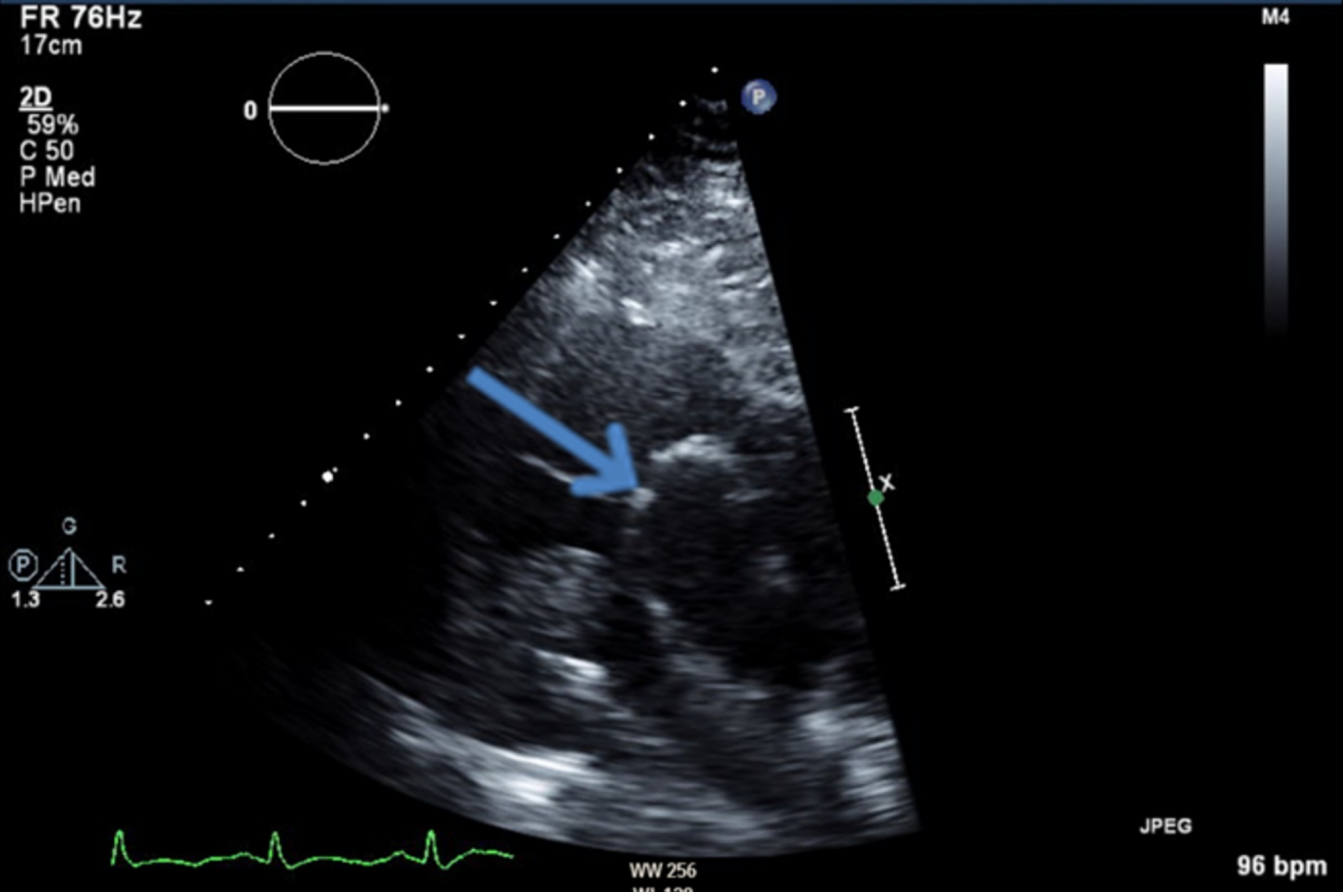
Echocardiography - short axis view at the aortic valve level showing the right atrial mass

**Figure 2:**
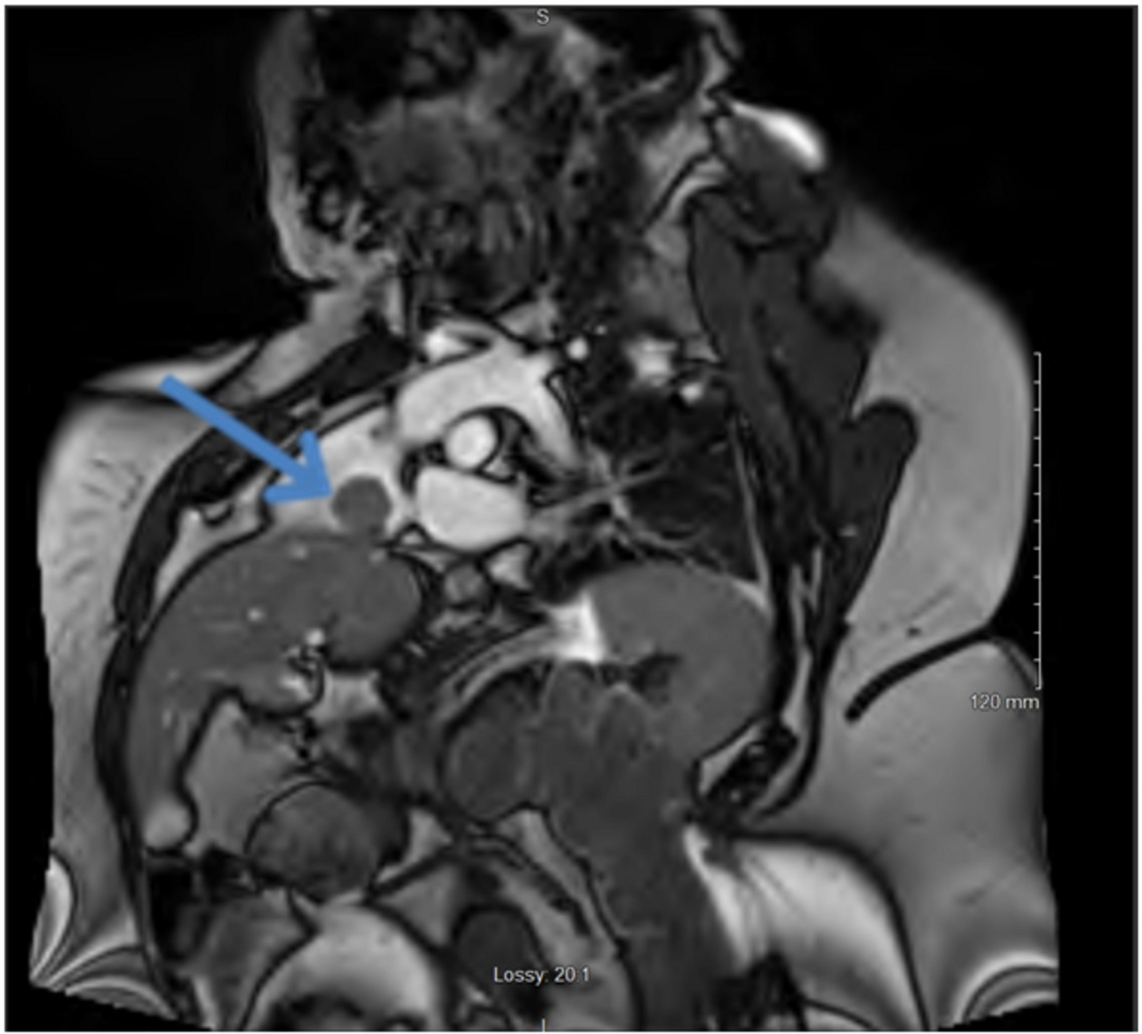
Cardiac magnetic resonance imaging (MRI) MRI image of cine sequence cutting through a right atrial mass showing the origin of the mass from the liver

**Figure 3:**
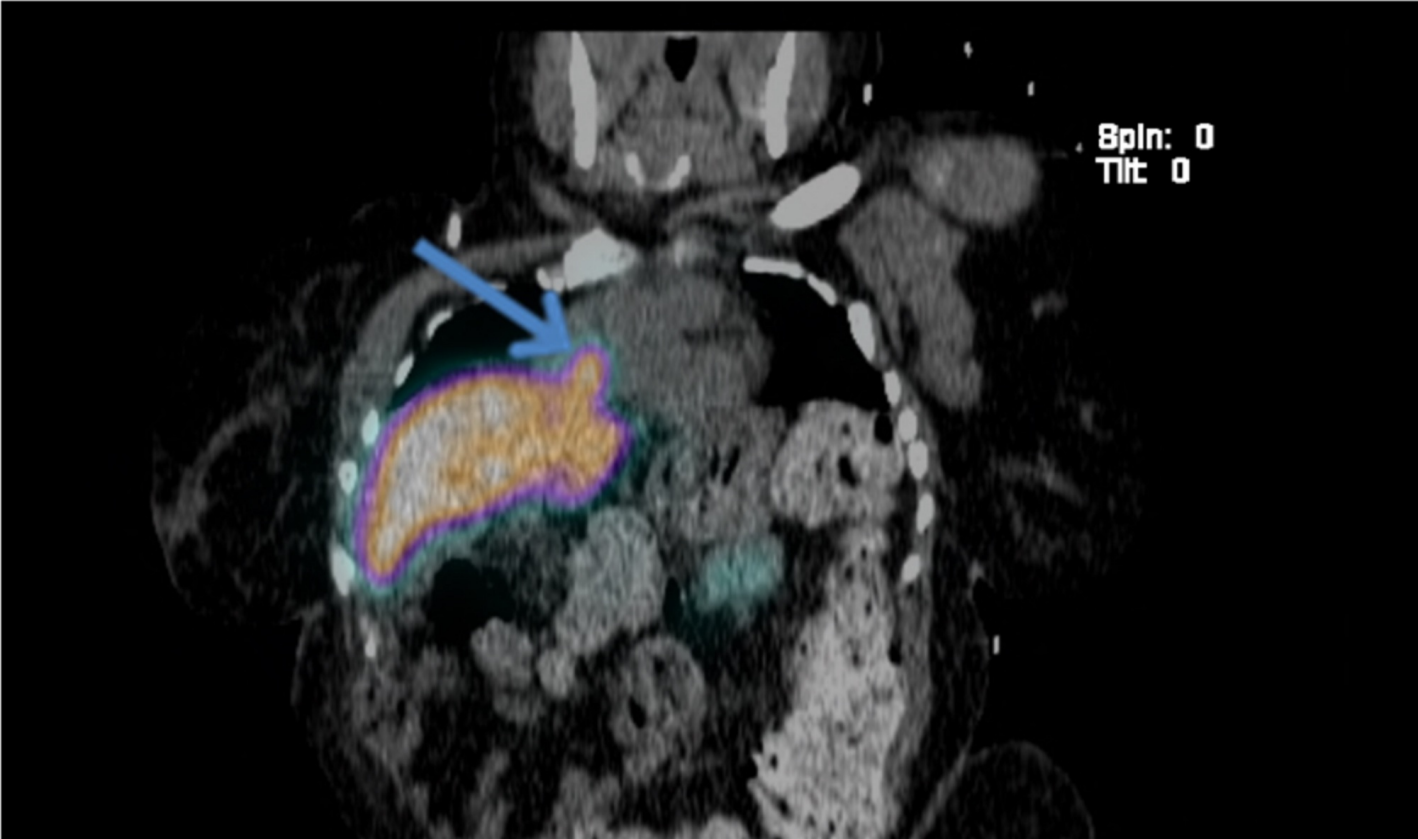
99m Tc-sulfur colloid SPECT/CT showing the liver and liver hernia uptake SPECT/CT: single photon emission computed tomography/computed tomography

The TTE showed a cardiac mass in the right atrium extending into the inferior vena cava (IVC) (Figure 1). For further identification of the mass, cardiac magnetic resonance imaging (MRI) was done which showed a normal left ventricular ejection fraction of 57%, right ventricular dilatation with moderately reduced systolic function of 36%, and a mass-like structure projecting over the right atrium with the appearance most suspicious for a diaphragmatic caval foramen herniation of liver parenchyma encroaching on the right atrium (Figure 2). A sulfur colloid nuclear single-photon emission computed tomography (SPECT) study also confirmed this finding (Figure 3). She continued inpatient treatment with anticoagulation and antibiotics during which she was seen by cardiology who recommended outpatient follow-up with cardiothoracic surgery for evaluation for potential surgical excision of the right atrial mass that was found to be herniated liver tissue.

## Discussion

The vast majority of intracardiac tumors are secondary to metastatic disease. Primary cardiac tumors are extremely rare and 75% of the time are benign. Myxoma is the most common primary cardiac tumor [4].

Intracardiac mass differential diagnosis is broad. It includes tumor, thrombus, vegetation, or foreign body. It also can be an extremely rare presentation of a heterotopic liver that has herniated through the vena cava into the right atrium, which can be mistakenly diagnosed as a right atrial tumor [3].

Ectopic liver is a rare condition that can be classified into four categories: (i) an accessory liver lobe attached to the liver, (ii) a large accessory liver lobe with a connecting stalk to the liver, (iii) an ectopic liver without connection to the liver, and (iv) microscopic ectopic liver tissue [4]. The present case is consistent with the second type of ectopic liver tissue. Typically, the presentation of aberrant hepatic tissue is in the abdominal cavity; however, it has been reported in the thoracic cavity [5]. There have been a few cases in which ectopic liver parenchyma has been described as a right atrial mass [6-8].

In the absence of trauma, the pathogenesis of heterotopic liver tissue is still unclear. Controversial theories have been documented in an effort to explain this liver tissue aberrancy. One theory proposed that it is a congenital defect of the septum transversum, embryonic tissue that differentiates into both the diaphragm and ventral mesentery of the foregut [1]. However, authors of another case report hypothesized that there can be hematogenous migration of hepatic cells with regenerative capacity even after intrauterine development. They supported their hypothesis with the absence of a detectable right atrial mass on TTE performed 18 months prior to the patient's presentation [9]. 

Although this ectopic liver tissue was discovered as an incidental finding during TTE, it can have serious clinical implications. Chapman-Fredrick et al. described a case of inferior vena cava (IVC) obstruction by a hernia liver mass [10]. This can lead to downstream venous congestion and a higher risk of thromboembolic complications. Another clinical implication is the malignant transformation of aberrant liver tissue. Matsuyama et al. reviewed 100 cases of aberrant hepatic tissue and reported that 28 patients were found to have hepatocellular carcinoma on a pathologic review of the tissue [11]. This potential risk of carcinogenesis should increase the urgency for closer follow-up, surgical excision with negative margins, and a careful pathologic review of the ectopic tissue.

Most cardiac masses are found incidentally during routine cardiac imaging. TTE is most commonly used, due to its affordability, portability, widespread availability, lack of exposure to ionizing radiation, and ability to provide anatomical and functional information of the heart. However, there are pitfalls to TTE. It may not be able to differentiate herniated liver masses from an atrial myxoma, secondary malignant masses, and in some cases, thrombus or vegetations. These limitations are resulting from the poor acoustic window, being operator-dependent, and having a narrower field of view [12]. Cardiac magnetic resonance imaging (MRI) might be of more value as it offers several advantages over TTE, such as a larger unrestricted field of view, superior soft-tissue contrast, operator independence, and consistent reproducibility [13]. Thus, cardiac MRI is a very helpful diagnostic tool when an intracardiac mass cannot be characterized by TTE.

## Conclusions

In exceedingly rare occasions, heterotopic hepatic tissue can present as a right atrial mass that can be mistakenly diagnosed as a right atrial myxoma or other more common etiologies of cardiac mass. Cardiac MRI should be the next step when the TTE study is not able to clearly characterize the right atrial mass. As this ectopic liver tissue carries a risk of malignant transformation, close monitoring and surgical excision with safety margins should be considered.
